# No Effect of Subthalamic Deep Brain Stimulation on Intertemporal Decision-Making in Parkinson Patients^1^,^2^,^3^

**DOI:** 10.1523/ENEURO.0019-16.2016

**Published:** 2016-05-23

**Authors:** Maayke Seinstra, Lars Wojtecki, Lena Storzer, Alfons Schnitzler, Tobias Kalenscher

**Affiliations:** 1 Comparative Psychology, Institute of Experimental Psychology, Heinrich-Heine University Düsseldorf, 40225 Düsseldorf, Germany; 2 Institute of Clinical Neuroscience and Medical Psychology, Medical Faculty, Heinrich-Heine University Düsseldorf, 40225 Düsseldorf, Germany

**Keywords:** deep brain stimulation, intertemporal choice, Parkinson’s disease

## Abstract

Deep brain stimulation (DBS) of the subthalamic nucleus (STN) is a widely used treatment for the motor symptoms of Parkinson’s disease (PD). DBS or pharmacological treatment is believed to modulate the tendency to, or reverse, impulse control disorders. Several brain areas involved in impulsivity and reward valuation, such as the prefrontal cortex and striatum, are linked to the STN, and activity in these areas might be affected by STN-DBS. To investigate the effect of STN-DBS on one type of impulsive decision-making—delay discounting (i.e., the devaluation of reward with increasing delay until its receipt)—we tested 40 human PD patients receiving STN-DBS treatment and medication for at least 3 months. Patients were pseudo-randomly assigned to one of four groups to test the effects of DBS on/off states as well as medication on/off states on delay discounting. The delay-discounting task consisted of a series of choices among a smaller. sooner or a larger, later monetary reward. Despite considerable effects of DBS on motor performance, patients receiving STN-DBS did not choose more or less impulsively compared with those in the off-DBS group, as well as when controlling for risk attitude. Although null results have to be interpreted with caution, our findings are of significance to other researchers studying the effects of PD treatment on impulsive decision-making, and they are of clinical relevance for determining the therapeutic benefits of using STN-DBS.

## Significance Statement

To improve the quality of life of patients with Parkinson’s disease, it is important to uncover the cognitive side effects of deep brain stimulation of subthalamic nucleus. In this study, we show no effect of deep brain stimulation on altered impulsive decision-making, measured with a financial delay-discounting paradigm. Our study adds an important piece of information on the cognitive side effects of deep brain stimulation, although further studies are needed to verify our results.

## Introduction

Parkinson’s disease (PD) is characterized by a cell loss in substantia nigra and ventral tegmental area, leading to a reduced level of the neurotransmitter dopamine and abnormal functionality of the basal ganglia. The progressive loss of dopamine results in impaired motor functioning, such as bradykinesia, muscle rigor, and/or resting tremor, as well as in characteristic nonmotor symptoms, including depression and memory deficits. Deep brain stimulation (DBS) of the subthalamic nucleus (STN) is a widely used treatment for the motor symptoms of PD. STN-DBS is usually applied when conventional medication starts to become increasingly ineffective (Deuschl et al., 2006). Although STN-DBS has major benefits in reducing motor symptoms (Deuschl et al., 2006; Wichmann and DeLong, 2006), the side effects of STN-DBS on cognition are often less clear (Demetriades et al., 2011).

Several studies indicate that DBS affects neural activity in surrounding areas, thereby altering the activity of a whole network of brain structures (Chang et al., 2007; Li et al., 2007; McCracken and Grace, 2007; Montgomery and Gale, 2007; Li et al., 2012). Since the STN is connected to a number of basal ganglia nuclei as well as cortical areas, STN-DBS can have widespread effects that are not just limited to motor behavior. Not only motor areas are found to be projecting to the STN, but also brain areas involved in the valuation of choice options, such as the medial/orbital cortex in rats (Maurice et al., 1998) and monkeys (Haynes and Haber, 2013) via the so-called hyperdirect pathway (Nambu et al., 2002), which links the cortex with the basal ganglia via the STN. In addition, the STN can be subdivided into several functional zones that can, according to their structural connectivity, be identified as motor, associative, and limbic regions (Lambert et al., 2012), which are part of corticobasal ganglia-thalamo-cortical loops involved in emotion, movement, and cognition (Parent and Hazrati, 1995a,b).

Patients have often undergone a long period of dopaminergic medical treatment before DBS is considered as the therapy of choice. Dopaminergic treatment usually consists of the intake of levodopa (l-dopa), a dopamine precursor, and/or dopamine agonists. An increased tendency for impulse control disorders (ICDs), which include pathological gambling, compulsive shopping, hypersexuality, and hyperphagia (Weintraub, 2008), can develop in PD patients. These ICDs are associated with dopaminergic treatment, in particular with the use of dopamine agonists (Voon and Fox, 2007; Voon et al., 2011a,b; Raja and Bentivoglio, 2012) as well as l-dopa treatment (Zurowski and O'Brien, 2015).

How STN-DBS affects impulsive behavior is unclear, with reports of increases in both the severity of even the new development of ICDs (Hälbig et al., 2009; Lim et al., 2009; Broen et al., 2011; Moum et al., 2012), as well as the attenuation or disappearance of ICD symptoms after the start of STN-DBS treatment (Witjas et al., 2005; Ardouin et al., 2006; Bandini et al., 2007; Lim et al., 2009; Broen et al., 2011). As the dopaminergic medication intake can usually be decreased after the onset of STN-DBS treatment, the reduction in ICD severity might be due to a decrease in the medication dosage, but other factors, such as electrode placement, stimulation parameters, or patient history may underlie changes in ICD severity too (Zurowski and O'Brien, 2015). Several brain areas connected with the STN are involved in impulsive behavior, including the orbitofrontal cortex and the nucleus accumbens (Cardinal et al., 2001; Kheramin et al., 2002; Kalenscher and Pennartz, 2008). Stimulation of the STN can therefore affect impulsive choice in the following two ways: either by directly altering STN functioning, and/or via indirect moderation of activity in connected areas known to be involved in impulsive decision-making.

Since (case study) reports concerning the effects of therapeutic STN-DBS on ICDs are ambiguous, it is important to uncover exactly how STN-DBS affects impulsive behavior, and in particular impulsive choice. The study presented here focuses on delay discounting (i.e., the devaluation of a reward when its receipt is delayed to a future point in time), which can be seen as a measure of impulsive economic decision-making, and is often used to assess impulsive decision-making (Bickel et al., 2012). Although delay discounting captures only one of the many facets of ICDs, reduced delay sensitivity lies at the heart of most concepts of impulsive choice. To dissociate the putative effects of STN-DBS from the effects of dopaminergic medication on delay discounting, we used a 2 × 2 design for DBS (on/off) and medication state (l-dopa on/off).

## Materials and Methods

### Participants

Fifty-four patients with bilaterally implanted stimulation electrodes in the STN were recruited for a screening session at the University Clinic Düsseldorf (Center for Movement Disorders and Neuromodulation, Department of Neurology, Institute of Clinical Neuroscience and Medical Psychology, Heinrich-Heine University Düsseldorf), with the aim of identifying patients with no current severe depression [Beck Depression Inventory (BDI), <20], no indication of dementia [Mattis Dementia Rating Scale (MDRS), >130], and inconspicuous performance in a range of other cognitive and mnemonic tests (see below) for inclusion in the experiment. Forty patients (16 female) between 42 and 78 years of age (mean, 62.7 years of age; SD, 7.4 years of age) met the inclusion criteria. Further inclusion criteria were bilateral DBS of the STN for a period of at least 3 months and no preimplant history of major depression.

DBS treatment consisted of bilateral 130 Hz stimulation, except for two patients who received 174 Hz stimulation in the right hemisphere and 130 Hz stimulation in the left hemisphere, two patients who received bilateral 150 Hz stimulation, and one patient who received unilateral (right) 130 Hz stimulation. Stimulation intensity was either fixed on voltage (*N* = 26) or amperage (*N* = 14), with voltages ranging between 1.2 and 4.0 V and amperage ranging between 1.1 and 3.4 mA. Pulse width was set at 60 μs, with the exception of three patients receiving 62 μs pulses and one patient receiving 65 μs pulses. One patient received 60 μs in the left hemisphere and 90 μs in the right hemisphere. The average time since DBS implantation was 30.0 months (SD, 23.7 months), with a minimum of 3 months and a maximum of 85 months. All but one patient received dopamine replacement therapy, with an l-dopa equivalent dose (LED) ranging from 120 to 1975 (mean, 675; SD, 390). All participants were recruited within a time period of 16 months, during their periodic inpatient visits that lasted at least 2 nights. The year of diagnosis ranged from 1989 until 2012. All participants were instructed in detail about the experimental procedure as well as the payment procedure before they provided written informed consent. The study was approved by the local ethics committee of the Medical Faculty of the Heinrich-Heine University Düsseldorf.

### Materials

During screening, patients performed a range of tests designed to measure mood as well as cognitive and mnemonic traits [MDRS, BDI-II, Quick Delay Questionnaire (QDQ), Baratt Impulsiveness Scale (BIS), South Oaks Gambling Screen (SOGS), and Ardouin Behavior Scale (ABS); see below], along with a delay-discounting task [intertemporal choice task (ICT)], risk attitude measurements (Holt-Laury task), and motor skills assessment [Unified Parkinson’s Disease Rating Scale **(**UPDRS)] during testing sessions. We used the following tests.

#### Mattis Dementia Rating Scale

The MDRS was used to test for cognitive deficits (Mattis, 1988). This test is commonly used in clinical settings for older patients and can detect dementia disorders such as Alzheimer’s disease. It is subdivided into the following five categories: attention, verbal and motor initiation and preservation, construction, conceptualization, and memory (Lucas et al., 1998). Patients with scores of <130 points (of a total of 144 points) were excluded from further testing (Schmidt et al., 1994).

#### Beck Depression Inventory II

The German version of the BDI-II (Beck et al., 1996) was used to assess depressive symptoms reported for the previous 2 weeks. It consists of 21 items, and each item is ranked from 0 to 3. The exclusion criterion was a count of ≥20 points, which is indicative of severe depression.

#### Quick Delay Questionnaire

The QDQ was administered to assess subjective delay aversion and delay discounting (Clare et al., 2010). The subjects have to rate five items on delay aversion and five items on delay discounting on a 5-point Likert scale. This questionnaire was added to obtain a baseline self-reported measure of delay discounting/delay aversion.

#### Barratt Impulsiveness Scale

The BIS is often used as a measure of impulsivity, and its short German version (BIS-15; Spinella, 2007) has been used in the current study. Fifteen items assess nonplanning, motor, or attention impulsivity (Spinella, 2007). Each item is rated on a 4-point Likert scale. This questionnaire was added to obtain a baseline self-reported measure of impulsiveness.

#### South Oaks Gambling Screen

The SOGS (Lesieur and Blume, 1987) consists of 20 items and is commonly used to screen for pathological gambling. In this test, a score of ≥5 is considered as probable pathological gambling. This questionnaire was added to identify and control for problem gambling or gambling tendencies, respectively.

#### Ardouin Behavior Scale

This scale was designed to detect changes in mood and behavior in PD patients (Ardouin et al., 2009). This semi-structured interview entails 18 items and is rated in 5 points, from 0 (absent) to 4 (severe). The ABS was used to identify potential addictive tendencies (regarding food or medication intake) that might hint at an ICD.

#### Unified Parkinson’s Disease Rating Scale

Part III of the Movement Disorder Society-sponsored revision of the UPDRS (MDS-UPDRS-III) was used to assess the severity of motor impairment, as well as the efficacy of the different treatment states. Patients had to perform specific movements and were rated from 0 to 4 on each of 18 items covering tremor, rigidity, posture, agility, and general movement (Goetz et al., 2008). The MDS-UPDRS-III was used to assess differences in motor symptoms between the respective on/off states during sessions.

#### Intertemporal Choice Task

**Figure 1.**
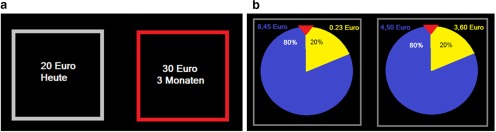
Screenshot of tasks. ***a***, Intertemporal choice task. Participants chose between a smaller reward now or a larger reward later by pressing the E or I key. When the choice was made, the chosen option was highlighted by a red frame. ***b***, Holt-Laury task: participants chose one of two gambles, one considered risky and one considered safer. Lotteries were depicted as wheels of fortune.

The ICT used in this study is a common and well validated task with which to elicit time preferences and measure delay discounting (Kirby and Maraković, 1996; Hardisty et al., 2013). The task consisted of a series of binary choices between a smaller, sooner, and a larger, later monetary reward. Choice items were arranged in six blocks with 11 trials each, with an instruction screen after each block to provide the opportunity to take a short break. Within each block, the amount of the smaller, sooner option varied over trials, while the larger, later option remained constant across trials within a given block. The delays used within each block were specified in the instruction screen before each block. In three blocks, the larger, later reward was fixed at €20, with the smaller, sooner option ranging from €0 to €20 in steps of €2, presented in randomized order. In the other three blocks, the larger, later reward was fixed at €30, with the smaller, sooner option ranging from €0 to €30 in steps of €3, presented in randomized order. The smaller, sooner option was always immediate. For each of the two large reward amounts, the delay was 3, 6, or 9 months, and the order was randomized across blocks. The options were presented simultaneously on the left and right sides of the screen, and the side of presentation of each choice option was randomized (Fig. 1*a*). Participants pressed the “E” key to choose the left option and the “I” key to choose the right option. There was no time limit for each choice. The trials with either €0 “now” or €20/€30 now were considered catch trials, as the choices in these trials indicate whether the participant paid attention or chose rationally. The task was programmed and conducted using the MATLAB (MathWorks) toolbox *Cogent*. One of the 66 trials was randomly chosen for payment after task performance. Participants received the amount they had selected with the corresponding delay. Both immediate and delayed payment was accomplished by a check that was given either right after the session (immediate payment) or was sent by mail (delayed payment).

#### Holt-Laury task

The Holt-Laury task (Holt and Laury, 2002) is a short, thoroughly validated 10-trial task to measure risk attitude (Filippin and Crosetto, 2014). Here, we elicited risk attitude as a control variable as time preference measures may potentially be confounded with risk preference. In each trial, participants chose between two lotteries. In one of the lotteries, the payout was either €8.45 or €0.23 with variable probability (riskier lottery); in the other lottery, the payout was either €4.50 or €3.60 with the same variable probability (safer lottery). The probability of winning the large reward of each lottery varied from 10% to 100% in steps of 10% across trials in randomized order. Correspondingly, the probability of winning the small reward was 100% − *p*(large reward). The probabilities of large and small rewards were identical for both lotteries in a given trial (Fig. 1*b*). After task performance, the computer randomly picked one trial and played the lottery that was chosen. The outcome was paid by check at the end of the session.

### Procedure

PD patients were recruited and tested during their regular visit to the clinic, which lasted at least 2 nights. After patients were informed about the procedure of our experiment and provided written informed consent, they underwent the screening session in the afternoon on the day of their arrival, or 1 d after, at the clinic. The screening session involved the mood, memory, and cognition tests outlined above, and lasted ∼1 h. During screening, patients were always in their most optimal treatment state (i.e., on-stimulation and on-medication).

To test the effect of DBS and l-dopa on delay discounting, we used a between-subject 2 × 2 design with the factors medication (medication on vs off) and STN-DBS (on vs off). Forty patients were randomly assigned to one of the four treatment groups (10 patients/group). The testing procedures were as follows.

A regular visit included an ∼16 h period in which patients refrained from taking medication on either the first or the second night of their stay, starting at about 8:00 P.M. If the test session took place in the on-medication state, patients received 1.5× their regular dose of l-dopa (but never more than the maximum dosage of 200 mg), and/or other medication (dopamine agonists; see Table 4), on the morning of the test session, 1 h before the start of the session, to ensure a robust on-state during the whole procedure. Off-medication testing was always performed in the morning after spending a night without medication.

**Figure 2.**
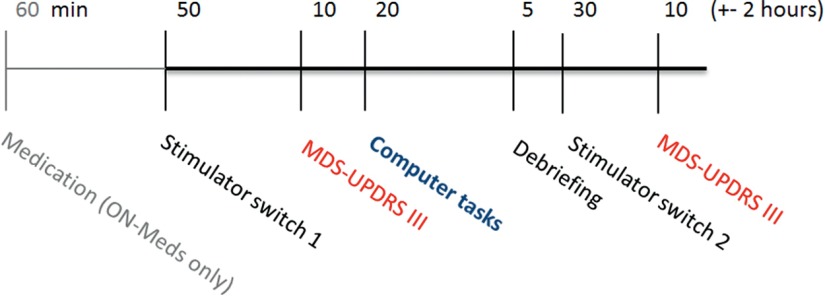
Schematic overview of a session. If patients were tested in the on-medication condition, they received medication (1.5× their regular l-dopa dose) 60 min before DBS was switched off or left on. Patients in the off-medication condition had not ingested dopaminergic medication since the previous evening. At the end of a session, a second MDS-UPDRS-III assessment was conducted in the opposite DBS state to confirm DBS effects within subjects.

A test session (for overview, see Fig. 2) took place between 9:00 A.M. and noon, and was conducted by two experimenters, of whom only one knew the current DBS state of the patient (passive experimenter), and the other exclusively interacted with and guided the patient through the session (active experimenter). The test sessions started with switching the DBS state of the patient. To ensure double blindness regarding the DBS state, the stimulator was either turned off or left on by a nurse or doctor who was informed by the passive experimenter, without informing the patient about what was done. The patient was aware that the stimulator would be either turned off or remain on and was informed beforehand about the necessity of the double-blind procedure. At least 50 min after the switch, the MDS-UPDRS-III was conducted, followed by the delay-discounting task (ICT) and subsequently the Holt-Laury risk attitude task. Each patient received oral instructions before each task, and was asked control questions to ensure that they understood the tasks. The MDS-UPDRS-III, ICT, and Holt-Laury tasks were completed in ∼30–40 min. Several trials in the tasks were randomly selected for payout (see above). The patient received feedback about the trials chosen for payment immediately after completing the two tasks and was paid accordingly by means of a check. Directly after, the patient was asked about his/her strategy during the choice tasks and was informed about the goal of the experiment. Thirty minutes after changing the stimulation state, a second motor assessment using the MDS-UPDRS-III was conducted as a within-subjects control of the DBS state. A within-subjects repetition of the ICT and Holt-Laury task was not conducted because both tasks were deemed to be unsuitable for repeated measures within the short timeframe of one or two mornings.

### Data analysis

We used a 2 × 2 between-subjects factorial design with medication (on vs off) and DBS state (on vs off) as independent factors, and choice parameters (see below) as a dependent variable. To estimate discounting parameters in the ICT, we used the following two different, well established models: the hyperbolic discounting model (Mazur, 1984); and the Laibson (1997) quasi-hyperbolic discounting model (see below). In addition, we also used the total number of choices of the smaller, sooner option as a model-free measure of discounting (yielding a value between 0 and 66), as well as a model-free measure of present bias (i.e., the overweighting of immediate outcomes, see below for details). For the Holt-Laury task, we used the switching point [i.e., the probability at which the participant was indifferent between the two gambles (Holt-Laury task indifference points [HL-IPs])]. This measure was obtained using logistic regression. A higher switching point indicated more risk aversion.

#### Fitting of discounting models

All mathematical procedures to determine the participants’ discount parameters were performed using MATLAB (MathWorks). We first identified the individual IPs (the magnitude of the smaller, sooner reward that renders it equally valuable to the larger, later reward) for each of the six blocks, using logistic regression. This resulted in three values between 0 and 20 for the three blocks with €20 as maximum reward, and three values between 0 and 30 for the three blocks with €30 as the maximum reward.

We first fitted the standard hyperbolic model separately to the IPs of blocks 1–3 and blocks 4–6, using the following equation (Mazur, 1984):

(1) $${\text{SV}}_{\text{T}}=\text{A}/\left(1+k\text{T}\right)$$,

where *SV* is the subjective value of the reward at delay *T* (in months), *A* is the monetary amount of the reward, and *k* is the hyperbolic discount parameter describing the steepness of the discount function. The amount was set to *A* = 1 as the values were expressed as proportions of the later reward. Larger *k*-values indicate a greater impact of delay on value and therefore steeper discounting. The resulting *k*-values for the €20 and €30 blocks were subsequently log transformed and averaged to obtain one *k*-value per individual (note that the correlation between the two *k*-values for the €20 and €30 blocks was very high; *r* = 0.96, *p* < 0.000).

Further, the Laibson quasi-hyperbolic β-δ model was separately fitted to the indifference points of blocks 1–3 and 4–6 to obtain measures of present bias and patience, as follows:

$$SV_{T=0}=1$$

(3) $${\text{SV}}_{\text{T}>0}=\beta \times \delta^{\text{T}}$$.

*SV_t_* is the subjective value of a reward at time *T*. This equation models the often observed initial rapid decline in subjective value with small delays (present bias) separately, represented by the parameter β (with 0 ≤ β ≤ 1). The inverse of β can be interpreted as the extra weight added to immediacy, thus smaller β values can be construed as stronger present bias. The discount rate of the discount function is log(1/δ). Thus, the parameter δ (with 0 ≤ δ ≤ 1) can be interpreted as a measure of patience, with higher δ values indicating higher patience. The resulting β and δ parameters for the €20 and €30 blocks were subsequently averaged to obtain one β and δ value for each participant [note that there was a strong correlation between the β values of the €20 and €30 blocks (*r* = 0.83, *p* < 0.000) and the δ values (*r* = 0.59, *p* < 0.000)].

The model fits were performed for each participant individually, using a least-squares algorithm implemented in MATLAB R2013a (MathWorks). The fitting parameters *k*, β, and δ were allowed to vary freely. We calculated the Akaike Information Criterion (AIC) for each model per participant to check the goodness of fit of each model. We then averaged the scores across all participants, resulting in one average AIC value for the hyperbolic model and another AIC value for the Laibson quasi-hyperbolic model. These AIC scores showed that, in general, the data were better described by the quasi-hyperbolic model (mean, −17.5) than the standard hyperbolic model (mean, −10.1). However, when comparing individual AIC values, the quasi-hyperbolic model had higher AIC values compared with the hyperbolic model in 10 participants, indicating a better fit of the hyperbolic model in these participants.

To obtain an additional, model-free measure of present bias, we used the following formula:

Present bias (PB) = (large reward − 3 months IP)/(6 months IP − 9 months IP). To obtain an overall measure, we averaged the model-free present bias measure for the €20 and €30 blocks (PB). A higher score indicated more present bias.

### Statistical analysis

The statistical analyses reported below were performed using the IBM software package SPSS Statistics 20. We mainly used standard ANOVAs and ANCOVAs to investigate the main effects of DBS and medication state, as well as their interaction on the dependent variables described above. When necessary, we selected the Gabriel pairwise comparisons test as the *post hoc* test, which is robust against differences in group sample size. Furthermore, we used Bayesian statistics (Wagenmakers, 2007; Masson, 2011) to calculate the evidence in favor of the null hypothesis.

## Results

### Subject demographics and trait variables

Data from eight participants were excluded as they chose the dominated alternative on >6 of the 12 catch trials in the ICT (i.e., they selected €0 now over €20/€30 later; or they selected €20/€30 later over the same reward now; see above). In addition, two of these participants scored ≥5 points on the SOGS, indicating potential pathological gambling behavior. Our results do not change when these subjects are included in our analysis, except when explicitly mentioned below. Table 1 shows the general descriptive statistics of the remaining 32 patients. The DBS-on group consisted of 18 participants, of whom 8 were tested in the on-medication state. The DBS-off group consisted of 14 participants, of whom 7 were tested in the on-medication state. There was no significant difference in any of the demographic parameters between DBS and medication groups, except for age (*F*_(3,28)_ = 3.00, *p* = 0.047, η^2^ = 0.24; Table 1).

**Table 1.** Demographic, screening and questionnaire results per DBS/Med state

	**State (MED/DBS)**				**Statistics**	
	**(1) On/On** **(*N* = 8)**	**(2) On/Off** **(*N* = 7)**	**(3) Off/On** **(*N* = 10)**	**(4) Off/Off** **(*N* = 7)**	***F* (*p* value)**	***Post hoc* test (Gabriel)**
Age (years)	66.5 (1.4)	57.1 (1.4)	63.5 (2.6)	64.7 (2.9)	3.00 (0.047)*	Group 1 vs 2: *p* = 0.045*
Year diagnosis	2001 (2.3)	2001 (2.0)	2000 (2.2)	2000 (2.0)	0.09 (0.963)	
Months receiving DBS	30 (8.6)	20 (5.1)	30 (8,6)	39 (10.0)	0.75 (0.534)	
LED	594 (209.4)	671 (118.0)	623 (120.3)	642 (125.0)	0.04 (0.988)	
MDRS	139 (1.2)	138 (1.6)	138 (1.1)	138 (1.3)	0.19 (0.902)	
BDI	6.1 (1.4)	8.4 (1.5)	7.9 (1.2)	7.0 (1.0)	0.60 (0.620)	
BIS-total	25.8 (1.7)	32.3 (1.9)	32.5 (1.7)	25.0 (2.5)	4.34 (0.012)*	Group 3 vs 4: *p* = 0.055
BIS-nonplanning	9.3 (1.0)	11.9 (0.5)	11.5 (1.2)	8.1 (1.1)	2.74 (0.062)	
BIS-motor	8.9 (1.1)	10.0 (1.3)	11.1 (0.6)	8.4 (0.8)	1.66 (0.197)	
BIS-attention	7.6 (0.9)	10.4 (1.0)	9.9 (0.6)	8.4 (0.8)	2.54 (0.076)	
QDQ-total	22.9 (2.5)	24.9 (2.0)	26.0 (1.8)	20.3 (2.1)	1.40 (0.264)	
QDQ-discounting	11.1 (1.4)	12.0 (1.1)	12.5 (1.0)	10.6 (1.5)	0.48 (0.698)	
QDQ-Aversion	11.8 (1.3)	12.9 (1.5)	13.5 (1.6)	9.7 (1.3)	1.30 (0.294)	

*p < 0.05.

Table 1 shows the descriptive statistics of the screening tasks and questionnaires. A one-way ANOVA showed a significant difference between the groups in the self-reported impulsiveness (BIS-total), *F*_(3,28)_ = 4.34, *p* = 0.012, η^2^ = 0.317. However, Gabriel *post hoc* tests showed no significant differences between groups: group 1 versus 2: mean difference = −6.54, *p* = 0.157; group 1 vs 3: mean difference = −6.75, *p* = 0.084; group 1 vs 4: mean difference = 0.75, *p* > 0.999; group 2 vs 3: mean difference = −0.21, *p* > 0.999; group 2 vs 4: mean difference = 7.50, *p* = 0.107; group 3 vs 4: mean difference = 7.50, *p* = 0.055. Nevertheless, we included BIS-total scores as a covariate in all subsequent analyses to account for potential group differences in impulsiveness. Note that all participants filled out the questionnaires in their optimal (on-medication, on-stimulation) state, so this difference in BIS-total scores reflects a trait difference between groups, not the effect of DBS on impulsiveness.

### Differential treatment effects on motor scores, but not delay discounting

**Figure 3.**
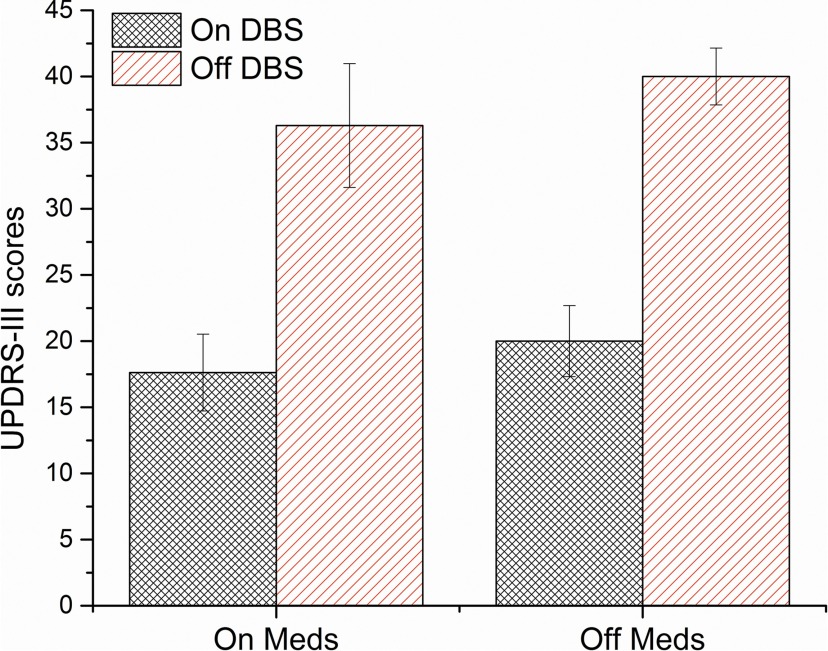
MDS-UPDRS-III scores for each DBS and medication state. Higher scores indicate greater motor impairments. Error bars show SEs.

As expected, MDS-UPDRS-III scores were significantly different between DBS/medication states, *F*_(3,28)_ = 11.96, *p* < 0.001, η^2^ = 0.56 (Fig. 3). *Post hoc* tests revealed a significant difference between DBS states (group 1 vs 2, 0.002; group 3 vs 4, *p* ≤0.001), whereas no significant difference was observed between medication states (group 1 vs 3, *p* = 0.993; group 2 vs 4, *p* = 0.990). This is likely due to relatively high interindividual differences in motor scores obscuring the relatively small but often beneficial effect of medication treatment within subjects. Comparing the MDS-UPDRS-III scores within patients (DBS on vs off only) also showed a significant improvement of motor symptoms with stimulation, time × DBS interaction (*F*_(1,31)_ = 138.84, *p* < 0.001, η^2^ = 0.82). Overall, this indicates that DBS significantly improved motor symptoms in our sample, while medication did not.

**Figure 4.**
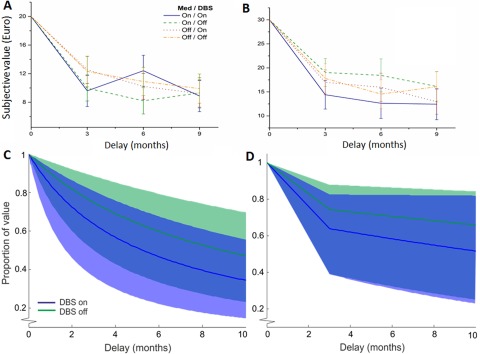
***A***, ***B***, Discounting curves per medication/DBS state subgroup for €20 (***A***) and €30 (***B***), based on the indifference point at 3, 6, and 9 months. Error bars show SEs. ***C***, Plots of the hyperbolic model in the on-DBS and off-DBS states, based on the median *k*-value. Shaded areas show the 25th and 75th percentile range. ***D***, Plots of the quasi-hyperbolic model in the on-DBS and off-DBS state based on the median β and δ values. The initial linear decline represents present bias and is determined by the β parameter, whereas the subsequent exponential curve represents “patience” and is determined by the δ parameter. Shaded areas show the 25th to 75th percentile range.

**Figure 5.**
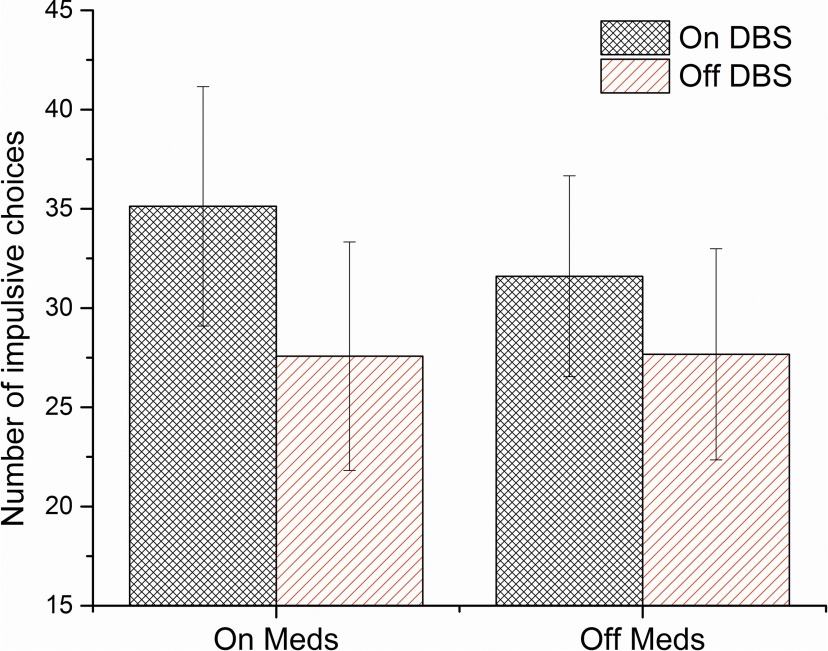
The total number of impulsive choices (smaller, sooner reward) for each DBS and medication state. Error bars show SDs.

Table 2 shows the discounting parameters *k*, β, and δ, the number of impulsive choices (NImp), the model-free measure of PB, as well as the HL-IPs within each group. We used a two-way ANOVA to test for the effects of DBS and medication on discounting and risk parameters, as well as on their interaction. We found no significant main or interaction effects of DBS or medication on any of the discounting parameters (Table 2). Figure 4, *A* and *B*, shows the discounting curves for each medication/DBS state for €20 and €30 blocks, respectively. Figure 4, *C* and *D*, shows the median fits of the hyperbolic and quasi-hyperbolic models, respectively, as well as the 25th and 75th percentile borders, for each DBS state. Figure 5 shows the total number of impulsive choices for each medication/DBS state. When adding age and the BIS-total score as covariates in an additional ANCOVA, THE main and interaction effects of DBS and medication states on any of the discounting parameters remained nonsignificant (DBS state: ln(*k*): *F*_(1,28)_ = 0.23, *p* = 0.636, η^2^ = 0.009; NImp: *F*_(1,28)_ = 0.41, *p* = 0.526, η^2^ = 0.018; β: *F*_(1,28)_ = 0.819, *p* = 0.37, η^2^ = 0.029; δ: *F*_(1,28)_ = 0.002, *p* = 0.967, η^2^ = <0.001; PB: *F*_(1,28)_ = 1.00, *p* = 0.325, η^2^ = 0.037; Table 2).

**Table 2.** Delay-discounting parameters and risk measure per DBS/Medication state

	**DBS**				**Medication**				**Interaction**	
	**On**	**Off**	**ANOVA**	**ANCOVAa**	**On**	**Off**	**ANOVA**	**ANCOVAa**	**ANOVA**	**ANCOVAa**
Ln(k)	−1.67 (0.38)	−2.17 (0.34)	0.90 (0.352)	0.23 (0.636)	−1.90 (0.33)	−1.88 (0.429)	0.003 (0.972)	0.09 (0.767)	0.18 (0.677)	0.13 (0.725)
NImp	33.2 (3.8)	27.1 (3.6)	1.31 (0.262)	0.41 (0.526)	31.6 (4.2)	29.5 (3.5)	0.17 (0.684)	0.46 (0.502)	0.053 (0.820)	0.24 (0.625)
βb	0.70 (0.08–1.0)	0.78 (0.35–0.98)	0.95 (0.338)	0.82 (0.374)c	0.62 (0.08–0.97)	0.80 (0.14–1.0)	1.25 (0.274)	1.55 (0.223)c	0.09 (0.765)	0.00 (0.999)c
δb	0.97 (0.83–1.0)	0.98 (0.78–1.0)	0.44 (0.511)	0.002 (0.967)c	0.99 (0.83–1.0)	0.97 (0.78–1.0)	1.19 (0.285)	1.21 (0.282)c	1.09 (0.306)	1.66 (0.208)c
PB	9.19 (1.60)	7.00 (1.34)	1.20 (0.283)	1.00 (0.325)	9.48 (1.86)	7.13 (1.19)	1.14 (0.295)	1.10 (0.303)	0.31 (0.580)	0.003 (0.956)
HL-IPs	41.5 (7.5)	46.5 (11.4)	0.22 (0.641)		49.3 (8.6)	38.7 (9.5)	1.24 (0.375)		5.29 (0.029)*	

Values are reported as the mean (SE), unless otherwise indicated.

^a^Age and BIS-total scores were added as covariates.

^b^Due to violation of normality, median (range) is shown instead of mean (SE). The rank transform procedure was used to test for main effects and interactions.

^c^A nonparametric equivalent of ANCOVA, as discussed in the study by Quade (1967), was used. Here the resulting *F* statistic and *p* value are shown.

*p < 0.05.

To calculate the probability that the null hypothesis (no effect of DBS on delay discounting) is true given our data (*p*(H_0_|D)), we used a Bayesian approach developed by Wagenmakers (2007) and also described in detail in a tutorial by Masson (2011). We used the Bayesian information criterion to calculate the posterior probability *p*(H_0_|D), with the assumption that the null and alternative hypotheses are equally likely. The results are presented in Table 3. We found *p*(H_0_|D) values ranging between 0.73 and 0.81, indicating positive evidence in favor of the null hypothesis, as suggested by Raftery (1995).

**Table 3.** Bayesian posterior probabilities for the hypothesis that there is an effect (H_1_), or for the hypothesis that there is no effect (H_0_), of DBS on discounting measures, given our data

	**NImp**	**Ln(*k*)**	**β**	**δ**
*p*(H_0_|D)	0.731	0.774	0.765	0.813
*p*(H_1_|D)	0.269	0.226	0.235	0.187

Some patients were treated with dopamine agonists instead of, or in addition to, l-dopa. As dopamine agonists are associated with impulsive behavior (Zurowski and O'Brien, 2015), we checked for differences between the DBS groups in the LED when considering only patients who receive dopamine agonists (LED agonists; Table 4). In each of the DBS groups, five patients used dopamine agonists, with no significant difference in LED agonist levels between groups (*U* = 110.50, *p* = 0.561, *r* = 0.13).

**Table 4.** Number of participants receiving dopamine agonists, and the LED agonists of the dopamine agonists used, per DBS group

	***N***	**LED agonists**	**Average LED agonists**
DBS on	5	595	119.0
DBS off	5	837	167.4

The Holt-Laury task was added as a control for the fact that impulsive behavior sometimes correlates with altered risk preferences (Kalenscher and Pennartz, 2008). There were no significant main effects of DBS or medication on Holt-Laury task scores (DBS state: *F*_(1,28)_ = 0.22, *p* = 0.641, η^2^ = 0.01; medication: *F*_(1,28)_ = 1.24, *p* = 0.275, η^2^ = 0.04), suggesting no effect of DBS and/or medication on risk attitude. Note, though, that we found a significant interaction effect of DBS and medication state on HL-IPs (*F*_(1,28)_ = 5.29, *p* = 0.029, η^2^ = 0.16). However, when using the complete sample of 40 patients, the interaction effect of DBS and medication state on HL-IPs failed to reach significance (*F*_(1,39)_ = 1.00, *p* = 0.325, η^2^ = 0.027). Note that a relatively large number of patients showed an inconsistent choice pattern (i.e., switching more than once between the risky and safe gamble), with 47,5% making at least one error (one more switch) and 30% having at least two errors, compared with the numbers mentioned in the original article on the Holt-Laury task (Holt and Laury, 2002), where only 13.2% of the participants made at least one error.

## Discussion

In this study, we aimed to investigate the effect of STN-DBS on impulsive decision-making, using a delay-discounting paradigm. We found no evidence for an effect of either STN-DBS or medication on delay-discounting behavior, a commonly used measure of impulsive choice. Although we found a significant effect of the interaction of DBS and medication state on risk aversion, this effect did not hold when all participants were included in the analysis. In addition, due to the relatively large number of errors the participants made in this task, we refrain from further interpretation of this finding.

Our findings are in line with a study by Torta et al. (2012), who investigated the effects of STN-DBS on delay aversion. Twenty-one PD patients with STN-DBS turned on and off (patients were off medication) performed the Cambridge Gambling Task, which measured both risk behavior and delay aversion, and filled out questionnaires assessing self-reported delay aversion, delay discounting, and impulsivity. The authors found no effects of stimulation on delay aversion or task behavior, although patients self-reported a higher feeling of impulsivity in the off-stimulation state. Thus, while increased levels of delay discounting have been associated with several impulse control disorders, such as substance abuse, attention deficit hyperactivity disorder, as well as pathological gambling and overeating (Bickel et al., 2012)—behaviors often shown by PD patients in response to their treatment—there is no evidence so far that STN-DBS alters delay discounting.

Although the development of ICDs is often attributed to side effects of dopaminergic medication (Voon and Fox, 2007; Voon et al., 2011a,b; Poletti et al., 2013), several studies point toward a potential role of STN-DBS in the development of ICDs in PD patients (Hälbig et al., 2009; Lim et al., 2009; Moum et al., 2012). However, it has been argued that the development of ICDs after STN-DBS onset may be an indirect consequence of disease history and treatment, as they may result from long-term alterations of frontolimbic structures, which are presumed to be involved in ICDs (Brewer and Potenza, 2008), due to disease progress and long-term medication use (Moum et al., 2012). Because ICDs themselves are considered to be chronic disorders, a short change in DBS state, as applied here, after several months of chronic stimulation might not be sufficient to uncover potential long-term effects leading to the development of ICDs. This would be in line with findings pointing at an increase in cognitive impulsivity reported by both patients and relatives 3 months after STN-DBS onset compared with a baseline taken before STN-DBS onset (Pham et al., 2015), but would be contradictory to the above-mentioned self-reported increase in impulsivity in a short-term off-state compared with scores in the DBS-on state (Torta et al., 2012). Although the motor effects of STN-DBS are often visible within minutes, cognitive effects of STN-DBS on impulsive decision-making might not be visible in the short term. For example, as reward learning seems to be affected by STN-DBS, perhaps experiences with rewards after STN-DBS onset influence subsequent choice behavior that could lead to the development of ICDs in a subgroup of patients. Future studies need to monitor long-term changes in delay discounting in particular, and impulsivity in general, after STN-DBS treatment onset.

Impulsivity itself is considered a multifaceted construct (Evenden, 1999; Kalenscher et al., 2006), with one subtype being defined as impulsive action (the inability to inhibit a prepotent response) and another subtype defined as impulsive choice (preferring a smaller, more immediate reward over a larger, more delayed reward; Winstanley et al., 2004; Kalenscher and Pennartz, 2008; Robinson et al., 2009). Motor impulsivity is commonly assessed with reaction time tasks, in which motor responses need to be inhibited either before (“waiting”) or during (“stopping”) execution, whereas choice impulsivity is often assessed with an intertemporal choice task, in which participants make repetitive choices between a smaller/sooner and larger/later (often monetary) reward. Several studies have dissociated the cognitive and neural bases of these two types of impulsivity (Winstanley et al., 2004; Van den Bergh et al., 2006; Broos et al., 2012). So far, studies have uncovered the effects of STN-DBS on motor impulsivity (Witt et al., 2004; Frank et al., 2007; Aleksandrova et al., 2013), which is in line with literature supporting the involvement of the STN in controlling the threshold for responding in situations with high conflict (i.e., when two choice options are relatively similar in value; Baunez and Robbins, 1997; Baunez et al., 2001; Desbonnet et al., 2004; Frank, 2006; Cavanagh et al., 2011). With regard to reward processing and decision-making, STN-DBS seems to mainly influence reward learning (Serranová et al., 2011; van Wouwe et al., 2011) and the evaluation of losses (Rogers et al., 2011), but, to the best of our knowledge, there is no evidence so far of an effect of STN-DBS on risky decision-making (Brandt et al., 2015).

One concern with our study is the small sample size, and, by consequence, the low statistical power. We cannot reject the possibility that we missed a small effect of STN-DBS on delay discounting because we lacked the statistical power to detect it. However, our Bayesian analysis showed positive evidence in favor of the null hypothesis. This suggests that the effect size is either very small or nonexistent. Therefore, we can conclude with some confidence that, if there were a short-term effect of STN-DBS on delay discounting, it would be miniscule and probably negligible.

Note that we started off with a small pilot experiment to check whether our task was suitable for repeated measures, as this would greatly increase power. However, we found that patients often made stereotypical, repetitive choices on subsequent repetitions of the task, which was supported by anecdotal remarks about their choice behavior and strategy (e.g., they would ask why they had to do the same task again; or they specifically commented on the fact that they would remember their choices in the previous task, and aimed to copy their own choices). For this reason, we opted against using a repeated-measures design.

Additionally, we would like to note that, although highly undesirable, underpowered statistics are frequently unavoidable in studies with clinical populations; due to the difficulty of finding a sufficient number of patients meeting the inclusion criteria, patient samples in medical studies are often smaller than desired. Nevertheless, despite the admittedly low power, we believe that our results are of significance to other scientists studying the effects of PD treatment on impulsive decision-making. To prevent the so-called “file drawer effect” (i.e., publication biases due to potentially informative studies ending up not being published due to nonsignificant findings; Sterling et al., 1995; Hopewell et al., 2009; Song et al., 2009), we would like to make our findings accessible to researchers interested in similar research problems.

In conclusion, we failed to demonstrate a significant effect of STN-DBS on delay discounting. Although an absence of evidence is not evidence of absence, calling for interpretative caution, this could potentially imply that STN-DBS effects on delay discounting do not exist. From a clinical perspective, this study provides evidence for a lack of negative cognitive side effects of STN-DBS in the form of altered intertemporal decision-making. Even if a small effect of STN-DBS on delay discounting existed, a risk of slightly altered decision-making likely does not weigh the same as the benefits of STN-DBS on motor functioning. Our findings, therefore, underscore the clinical safety of DBS-STN as a therapeutic treatment.

## Synthesis

The decision was a result of the Reviewing Editor Julie Andersen and the peer reviewers coming together and discussing their recommendations until a consensus was reached. A fact-based synthesis statement explaining their decision and outlining what is needed to prepare a revision is listed below. The following reviewers agreed to reveal their identity: Ilse Pienaar, Jiaxiang Zhang

Given the concerns of particularly Reviewer #1, a revision of the manuscript will be required in order to consider it for publication in eNeuro:

Reviewer #1: In this study, the authors examined the effects of STN-DBS on impulse control disorder in Parkinson's patients. I have several concerns with the paper, which prompts me to recommend a re-review of the paper, following substantial improvements by the authors:

Major concerns:

- Could the authors provide evidence for correct placement of the STN for the probes? I realise that post-mortem histological verification is not possible, but can imaging data be shown? DBS electrode placements are notoriously variable, which explains some of the variable effects noted in outcome.

- There is considerable variance in the time that patients have been receiving DBS, ranging from 3-85 months. Time since DBS was initiated is potentially a major factor that should be taken into account during the data analyses.

- For the battery of tests, the authors are required to cite studies that have made use of the individual tests, to justify the choice of test. In some, such as the Holt-Laury task, only the original test published by Holt and Laury themselves had been cited.

- If the minimum effect size that can be detected is greater than 25% (as stated by the authors), I would question whether strong data interaction effects can really be ruled out here. The authors should therefore state what the smallest effect size was that they could detect.

- This concern raises a major concern which the authors acknowledge themselves, namely that ICDs are a chronic disorder, and therefore a short interruption of DBS may not be an adequate control measure to effectively assess if DBS has the potential to alter expression of ICDs (line 360). Bearing this in mind, I feel its essential for the authors to provide a more persuasive argument for the current experimental design. This is vital, in light of the lack of data on the patients' test performance prior to initiation STN-DBS (baseline scores).

- The authors do not make fully clear whether they hypothesize that STN-DBS could worsen PD patients' cognitive symptoms or in fact potentially improve them, although the "significance statement" sways more towards the side-effects route. Either way, the neural mechanism for this should be explicitly explained, i.e. a glutamatergic hyperexcitation effect to the basal forebrain. Although the authors provide some information on this, i.e. from line 37, the explanation they provide becomes confusing when they refer to neural projections TO the STN (line 39). Surely a cognitive effect will be due to efferent projections from the STN (being stimulated) projecting to brain regions proven to be involved in impulse control?

Minor comments:

- Several stylistic mistakes, e.g. "to or reverse impulse control disorders" (line 7).

- The term "hyperdirect pathway" should be explained (line 41).

- Several statements require referencing, i.e. the sentence starting with "Dopaminergic treatment" (line 46). The same is true for the statement starting with "side-effects" (line 33).

- There should be consistency in the use of abbreviations, such as Parkinson's disease which has been abbreviated to PD, but the terms PD and Parkinson's disease continue to be used interchangeably throughout the manuscript. The same can be said for deep brain stimulation, abbreviated to DBS, but then referring back to the full term again in line 93.

- There is some repetition, such as the sentence starting with "for each" (line 135), with a similar statement that occurred earlier at line 108.

- The text does not refer to Figure 1.

- The resolution of the tables for presenting data is very poor and the text is too small to read.

- Analyses of the data should determine for a possible gender effect.

- In line 276 the authors state that they made use of a Gabriel post-hoc test. This test is little known, compared to a widely used test such as the Tukey test. The authors should therefore state the reasons for including this test rather than a more standard type.

- The full definition of ADHD should be given in line 350 before using the abbreviation.

- In relation to the abstract, I can add the following:

To fully understand the content of the abstract, readers require specialist, field-specific knowledge. This is exemplified by use of the term "delay discounting", with no definition given for this by the authors. I feel it's important to highlight the main limitation of the study in the abstract, namely the small sample size. In light of this, I feel that the statement by the authors that the study could be of "clinical relevance for determining the therapeutic benefits of using STN-DBS" is too strongly put, with far greater caution that is warranted by the authors of this study. Also, the statement that forty patients performed the task could be perceived as deceitful, as this sample size was divided into 4 groups, representing the different conditions of the test protocols. The statement in line 13 ("between smaller, sooner and larger, later monetary rewards") can easily be wrongly interpreted as meaning a financial reward given "sooner and later", due to the incorrect placement of commas to describe the two different options.

Reviewer #2: This study was conducted carefully . I wholeheartedly agree the author(s) view and approach to publish null results. But there are few issues in the current manuscript.

1. If the main research question here is the DBS effect on TD responses, it is not clear why patients were not tested ON and OFF DBS (and medication) in a repeated-measure design. The manuscript commented that the tasks "were not deemed suitable for repeated-measures" (line 134). I believe this is not true (see pubmed-19838863). If there are practical reasons that a repeated-measure design was impossible, this limitation should be noted.

2. The power analysis is problematic. First, it was not an "a priori calculation" (line 327), because the effect size was estimated from current samples. Second, it is circular (and sometimes misleading) to conduct post-hoc power analysis on null results to infer statistical power, as it often underestimate the perspective power. If the study aims to demonstrate "no effect of" STN-DBS (as in the title and discussion), it should be appropriate to use Bayesian ANOVA, reporting Bayes factors in supporting of the null.

3. Model fitting procedure is not clear. Were the reported AIC values averaged across all participants? Was the best model always superior to the other in all subjects? Since AIC favours the quasi-hyperbolic model than the hyperbolic model, subsequent analysis should focus on the model with best fit. What are the rationales to draw inferences from an inferior model?

4. Was any patients on DA agonists or other DA drugs? Some dopamine agonists have additional aversive effects (in combination of l-dopa), causing impulsive behaviour. A summary table of medication would be useful.

Minor points:

1. Table 1. Please include N per group

2. Table 2 contained all the information in Figure 3. Keeping one of them is sufficient.

3. Figure 4. It would be useful to show the actual model fit, averaged across subjects.
